# Exploring Stigma and Attitudes of Healthcare Workers Toward Mental Illness: Psychometric Evaluation Using Community Attitudes Towards the Mentally Ill Scale in Oman: A Single-Centre Study

**DOI:** 10.1192/bjo.2026.11195

**Published:** 2026-06-30

**Authors:** Marwa Al Abdali, Salim Al-Huseini, Muna Al Kalbani, Said Al-Kaabi, Samir Al-Adawi

**Affiliations:** 1 Al Masarra Hospital, Ministry of Health, Muscat, Oman; 2 BSMHFT, Birmingham, United Kingdom; 3 Muscat College, Muscat, Oman; 4 Sultan Qaboos University Hospital, Muscat, Oman

## Abstract

**Aims:**

There is limited research exploring healthcare workers’ (HCWs) attitudes toward individuals with mental illness within mental health settings in the Arabian Gulf countries. Given their central role in patient care, understanding HCWs’ perceptions is vital for improving mental health services. Oman’s universal healthcare system provides free access, and psychiatric care is delivered through a tiered referral structure from primary to tertiary facilities.

This study aimed to (1) assess HCWs’ attitudes toward people with mental illness (PWMI) in a tertiary psychiatric hospital in Oman and (2) evaluate the reliability and construct validity of the Community Attitudes toward the Mentally Ill (CAMI) scale in this context.

**Methods:**

A cross-sectional survey was conducted from March to August 2020 among HCWs at a tertiary psychiatric hospital. 315 HCWs of various nationalities completed the survey (response rate=55%). The survey included socio-demographic data and the CAMI scale, which measures attitudes across four subscales: Authoritarianism, Benevolence, Social Restrictiveness, and Community Mental Health Ideology. Data analysis included t-tests, ANOVA, regression, and Confirmatory Factor Analysis (CFA) using SPSS and AMOS software.

**Results:**

Presently used 30-item CAMI scale showed an acceptable internal consistency (Cronbach’s alpha: 0.62–0.91) and acceptable model fit in CFA. Authoritarian and socially restrictive attitudes were more prevalent among Omani nationals, HCWs with longer psychiatric tenure, and workers with personal histories of mental illness. In contrast, benevolence and positive community mental health attitudes were linked to Omani nationality, psychiatric experience, working by desire, and personal mental health experiences. Furthermore, male participants exhibited lower stigma levels than females.

**Conclusion:**

Findings highlight the role of culture, work experience, and personal history of mental illness in shaping HCWs’ attitudes toward PWMI. The validated CAMI scale supports future research and targeted intervention development. Culturally sensitive education programmes are essential to reduce stigma levels and encourage inclusive mental healthcare in Oman.

